# Examining Self-Reported Physical Activity Levels Among Kidney Disease Patients in the United States Using the Behavioral Risk Factor Surveillance System (BRFSS) Database: A Retrospective Study

**DOI:** 10.7759/cureus.71461

**Published:** 2024-10-14

**Authors:** Nithin Karnan, Monica I Idugboe, Shivaashish Karumanchi Anantha Venkata Sai, Riya K Shah, Sai Praneeth Chaparala, Pooja Patel

**Affiliations:** 1 Internal Medicine, K. A. P. Viswanatham Government Medical College, Tiruchirappalli, IND; 2 Internal Medicine, American University of Barbados, Bridgetown, USA; 3 Internal Medicine, Tagore Medical College and Hospital, Chennai, IND; 4 Internal Medicine, Smt. Nathiba Hargovandas Lakhmichand (N. H. L. Municipal Medical College, Ahmedabad, IND; 5 Internal Medicine, Gayatri Medical College, Hyderabad, IND; 6 Internal Medicine, Gujarat Medical Education and Research Society (GMERS) Medical College and Hospital, Valsad, IND

**Keywords:** brfss database, kidney disease, physical activity, socioeconomic status (ses), united states

## Abstract

Introduction: Kidney diseases have a gradual and subtle onset, which usually results in end-stage renal disease with patients requiring renal replacement therapy. Though pharmacotherapy plays a crucial role, integrating lifestyle modifications like increased physical exercise has been associated with significant improvement in health for kidney disease patients.

Aims: This study aims to retrospectively analyze the prevalence of self-reported physical activity among patients of kidney disease in the United States, based on demographic, socioeconomic, and healthcare access variables for the year 2021.

Methodology: Data was extracted from the Behavioral Risk Factor Surveillance System Web Enabled Analysis Tool (BRFSS WEAT) for the year 2021. Descriptive data, including numbers and percentages, was generated for each variable using cross-tabulations in the BRFSS WEAT. The data was stored in Microsoft Excel (Microsoft Corporation, Redmond, WA, USA), and statistical analysis was conducted using R version 4.3.1 (R Foundation for Statistical Computing, Vienna, Austria, https://www.R-project.org/). Statistical tests employed included the Chi-square test and Fisher’s exact test to ensure robust and reliable results.

Results: A total of 436,057 people participated in the BRFSS study. Out of this, 17,054 (39%) self-identified to the question “ever told you had kidney disease.” In the past month, 10,004 (58.7%) participants having kidney disease were involved in physical activity, whereas 7,050 (41.3%) were not. Also, the level of physical activity among kidney disease participants was highest in ages 18-25 (75.5%), male gender (63.6%), White non-Hispanic race (59.4%), patients having advanced education (64.2%), employed (72.6%), high income (82.1%), and having routine checkup within the past one year (8,957, 58.3%).

Conclusion: Kidney disease patients in the United States who self-reported physical activity were found to have statistical significance between various demographic, socioeconomic, and last routine checkup variables. Prospectively, interventions must be designed to increase physical activity among kidney disease patients with a focus on high-risk demographic groups like older adults, people with lower socioeconomic status, and people who don't get regular checkups.

## Introduction

Kidney diseases, or renal diseases, also known as nephropathy, are damage to the kidney. Depending on the anatomic location involved, different renal diseases can be categorized into parenchymal, interstitial, tubular, and glomerular. The most common types of renal diseases are acute kidney injury and chronic kidney disease (CKD). More than one in seven, or 15% of US adults, or 37 million people, are estimated to have CKD. Up to 90% of people with CKD are unaware that they have the disease. CKD has a very slow and subtle onset, which usually results in end-stage renal disease with patients requiring renal replacement therapy [1]. There are many causes for acute kidney injury, which can be particularly divided into three criteria: prerenal, renal, and post-renal. Prerenal indicates low-volume states like heart failure, cirrhosis, and nephrotic syndrome, which leads to decreased renal perfusion. Renal causes include nephrotoxic agents, trauma, infection, and inflammation. Any kind of obstruction, like nephrolithiasis, benign prostatic hyperplasia, renal cell carcinoma, and bladder carcinoma, are some of the post-renal causes. The major risks associated with CKD are anemia, osteoporosis due to secondary hyperparathyroidism, vitamin D deficiency, functional immobility, and cardiorespiratory dysfunction.

There are numerous strategies to retard the disease progression and lower the associated risks. Current approaches mainly involve lifestyle and dietary modifications like controlling blood pressure, blood glucose, other cardiovascular risk factors, and pharmacological interventions [1,2]. While pharmacotherapy plays a crucial role, integrating lifestyle modifications like increased physical exercise has been associated with significant improvements in health for kidney disease patients. With the decrease in renal function, CKD patients experience deteriorating cardiopulmonary function and associated muscle atrophy [1,3]. As kidney disease progresses, the worsening skeletal muscle dysfunction leads to functional immobility and loss of independence. To incorporate physical rehabilitation therapies and exercise counseling in treatment plans for CKD patients, it is essential to assess the patient's physical activity on a daily basis in order to gauge the risk of dysmotility [4].

Assessment of physical function in patients with renal diseases proves invaluable to study the further stratification and involvement of various treatment strategies to curate specific management plans according to different variables [5]. The main objective of this paper is to retrospectively review the self-reported physical activity levels in kidney disease patients and correlate the possible association or direct effect that the level of physical activity has among various groups of people with renal diseases over demographic, socioeconomic, and other variables.

## Materials and methods

A retrospective original research study was conducted using the Behavioral Risk Factor Surveillance System (BRFSS) database. Data was extracted on 25th June 2024. This study involved non-human participant research, as the BRFSS database contains deidentified, publicly available data. Therefore, no ethics committee approval was needed.

Data was extracted on 25th June 2024, focusing on the year 2021. The primary variables of interest were the disease variable "Not including kidney stones, bladder infection, or incontinence; were you ever told you have kidney disease?" and the physical activity variable "During the past month, any physical activity or exercises such as running, calisthenics, golf, gardening, or walking for exercise?"

The study included several control variables to adjust for potential confounders, ensuring a comprehensive analysis. Demographic characteristics were carefully considered, including age (18-24, 25-44, 45-64, 65+), gender (male, female), and race (White non-Hispanic, Black non-Hispanic, Hispanic, other). These categories allowed for a detailed examination of how different demographic groups might experience the variables of interest differently. Socioeconomic characteristics were also a critical part of the analysis. Education was categorized into two main groups: basic education (never attended school or only kindergarten, grades 1-8 (elementary), grades 9-11 (some high school), grade 12 or GED (high school graduate), and advanced education (college one year to three years (some college or technical), college four years or more (college graduate). Employment (EMPLOY1) was divided into employed (employed for wages, self-employed) and not-employed (out of work for one year or more, out of work for less than one year, a homemaker). Income was grouped into low income (<$50,000), middle income ($50,000-$150,000), and high income (>$150,000).

Additionally, healthcare access was assessed using the variable 'checkup,' which was divided into two main categories: within the past year (1-12 months ago) and more than a year ago or never (1-2 years ago, 2-5 years ago, 5 or more years ago, or never).

Descriptive data, including numbers and percentages, was generated for each variable using cross-tabulations in the BRFSS Web-Enabled Analysis Tool. The data was stored in Microsoft Excel (Microsoft Corporation, Redmond, WA, USA), and statistical analysis was conducted using R version 4.3.1 (R Foundation for Statistical Computing, Vienna, Austria, https://www.R-project.org/). Statistical tests employed included the Chi-square test and Fisher’s exact test to ensure robust and reliable results.

## Results

In the year 2021 in the United States, 436,057 people participated in the BRFSS study. Out of this, 17,054 (39%) self-identified to the question “ever told you had kidney disease (not including kidney stones, bladder infection, or incontinence).” These were thus considered to have kidney disease.

Table 1 shows the prevalence of physical activity in the study participants. It was observed that 10,004 (58.7%) participants with kidney disease were involved in physical activity in the month before data collection, whereas 7,050 (41.3%) were not. Among participants who did not have kidney disease, 319,687 (78%) were involved, and 99,316 (23.7%) were not involved in physical activity in the past month. The Chi-square and Fisher’s exact test suggests that there is a statistically significant association between physical activity level and self-reported kidney disease (p<0.001), thus indicating that physical activity is significantly associated with a lower prevalence of kidney disease amongst the participants.

**Table 1:** Prevalence of self-reported kidney disease (not including kidney stones, bladder infection, or incontinence) by physical activity level Values are mentioned in n (%). p-value <0.05 is significant.

Questions		During the past month, any physical activities, or exercises such as running, calisthenics, golf, gardening, or walking for exercise (EXERANY2)		P-value (Chi-squared test)
Ever told you had kidney disease (not including kidney stones, bladder infection or incontinence)	Yes/no	Yes	No	<0.001*
	Yes	10,004 (58.7%)	7,050 (41.3%)	
	No	319,687 (76.3%)	99,316 (23.7%)	

Table 2 shows the prevalence of self-reported kidney disease (not including kidney stones, bladder infection, or incontinence) by physical activity level based on the demographic characteristics of study participants. The level of physical activity among patients with kidney disease is highest in age group 18-25 (75.5%), male gender (63.6%), and White Hispanic race (59.4%). Based on demographic variables, physical activity was found to be significantly associated with a lower prevalence of kidney disease amongst the participants (p<0.05).

**Table 2:** Prevalence of self-reported kidney disease (not including kidney stones, bladder infection, or incontinence) by physical activity level based on demographic characteristics of study participants Values are mentioned in n (%). p-value <0.05 is significant.

Variables	Kidney disease (not including kidney stones, bladder infection, or incontinence)	N	Physically active	Physically not active	P-value (Fisher's exact test)
Age groups (10 years)					
18-24 years	Yes	155	117 (75.5%)	38 (24.5%)	0.009*
	No	25,791	21,581 (83.7%)	4,210 (16.3%)	
25-44 years	Yes	1,334	944 (70.8%)	390 (29.2%)	<0.001*
	No	104,497	85,259 (81.6%)	19238 (18.4%)	
45-64 years	Yes	4,875	2,896 (59.4%)	1,979 (40.6%)	<0.001*
	No	145,138	110,583 (76.2%)	34,555 (23.8%)	
65+ years	Yes	10,690	6,047 (56.6%)	4,643 (43.4%)	<0.001*
	No	143,577	102,264 (71.2%)	41,313 (28.8%)	
Gender					
Male	Yes	7,419	4,718 (63.6%)	2,701 (36.4%)	<0.001*
	No	195,068	153,844 (78.9%)	41,224 (21.1%)	
Female	Yes	9,635	5,286 (54.9%)	4,349 (45.1%)	<0.001*
	No	223,935	165,843 (74.1%)	58,092 (25.9%)	
Race					
White, non-Hispanic	Yes	12,788	7,592 (59.4%)	5,196 (40.6%)	<0.001*
	No	308,197	239,379 (77.7%)	68,818 (22.3%)	
Black, non-Hispanic	Yes	1,528	829 (54.3%)	699 (45.7%)	<0.001*
	No	31,049	21,890 (70.5%)	9,159 (29.5%)	
Hispanic	Yes	1,194	669 (56%)	525 (44%)	<0.001*
	No	37,004	25,589 (69.2%)	11,415 (30.8%)	
Other	Yes	1,156	686 (59.3%)	470 (40.7%)	<0.001*
	No	32,493	25,008 (77%)	7,485 (23%)	

Table 3 shows the prevalence based on the socioeconomic characteristics of study participants. The level of physical activity in patients with kidney disease was highest in advanced education (64.2%), employed (72.6%), and high income (82.1%). Based on socioeconomic characteristics, physical activity was found to be significantly associated with a lower prevalence of kidney disease for all education levels, employment, and income categories.

**Table 3:** Prevalence of self-reported kidney disease (mot including kidney stones, bladder infection, or incontinence) by physical activity level based on socioeconomic characteristics of study participants Values are mentioned in n (%). p-value <0.05 is significant.

Variables	Kidney disease (not including kidney stones, bladder infection, or incontinence)	N	Physically active	Physically not ative	P-value (Fisher's exact test)
Education level					
Basic education	Yes	6,127	2,995 (48.9%)	3,132 (51.1%)	<0.001*
	No	130,374	84,235 (64.6%)	46,139 (35.4%)	
Advanced education	Yes	10,860	6,975 (64.2%)	3,885 (35.8%)	<0.001*
	No	286,414	233,857 (81.6%)	52,557 (18.4%)	
Employment					
Employed	Yes	3,694	2,681 (72.6%)	1,013 (27.4%)	<0.001*
	No	219,434	178,387 (81.3%)	41,047 (18.7%)	
Not employed	Yes	13,132	7,199 (54.8%)	5,933 (45.2%)	<0.001*
	No	191,808	135,545 (70.7%)	56,263 (29.3%)	
Income					
Low income	Yes	8,172	4,298 (52.6%)	3,874 (47.4%)	<0.001*
	No	141,517	94,773 (67%)	46,744 (33%)	
Middle income $50,000 to $150,000	Yes	4,476	3,086 (68.9%)	1,390 (31.1%)	<0.001*
	No	149,953	124476 (83%)	25,477 (17%)	
High income >$150,000	Yes	733	602 (82.1%)	131 (17.9%)	<0.001*
	No	37923	34,535 (91.1%)	3,388 (8.9%)	

Table 4 shows the prevalence based on time since the last routine checkup. Among participants with routine checkups within the past one year, 8,957 (58.3%) participants with kidney disease were physically active as compared to the physically inactive participants (6404, 24.4%). Based on demographic variables, there is a statistically significant association between physical activity and kidney disease. Among participants with routine checkups for more than a year or never, 955 (63.1%) participants with kidney disease were physically active as compared to the physically inactive (558, 36.8%).

**Table 4:** Prevalence of self-reported kidney disease (not including kidney stones, bladder infection, or incontinence) by physical activity level based on time since last routine checkup Values are mentioned in n (%). p-value <0.05 is significant.

Variables	Kidney disease (not including kidney stones, bladder infection, or incontinence)	N	Physically active	Physically not active	P-value (Fisher's exact test)
Time since the last routine checkup					
Within the past one year	Yes	15,361	8,957 (58.3%)	6,404 (41.7%)	<0.001*
	No	319,911	241,994 (75.6%)	77,917 (24.4%)	
More than a year ago or never	Yes	1513	955 (63.1%)	558 (36.9%)	<0.001*
	No	93,591	73,969 (79%)	19,622 (21%)	

Irrespective of the time since the last routine checkup (within the last one year or more than a year ago), physical activity in the last one month is significantly associated with a lower prevalence of kidney disease among the participants. Based on demographic variables, there is a statistically significant association between physical activity and kidney disease.

## Discussion

A retrospective study was done in July 2024 to analyze the physical activity levels among patients with self-reported kidney disease in 2021 in the United States using the BRFSS database.

Physical activity has proven beneficial in improving the quality of life and reducing the progression of kidney disease. However, it has been misinterpreted that physically active patients with CKD may have increased proteinuria affecting kidney function [6]. Various randomized controlled trials and observational studies concluded that physical activity lowers blood pressure, reduces the progression of the disease, and lowers mortality due to cardiovascular disease [7]. In a narrative review, it is assessed that CKD accelerates muscle death and reduces muscle protein production due to the uremic state in the body. This process can be reversed with lifestyle interventions like physical activity and diet [8]. In a randomized controlled trial, 46 patients with severe CKD were analyzed, and the study group executed aerobic exercises thrice weekly and resistance training twice weekly for a period of six months. It was included that aerobic exercises improved the quality of life and reduced inflammation, leading to improvement in renal function without any negative impact on the patients [9]. A meta-analysis consisting of 2,156 participants from 35 research articles analyzed the factors of physical activity like time spent and difficulty levels of exercise beneficial in reducing the progression of the disease. It was concluded that an increased amount of time spent on these activities can be effective in improving the outcomes [10].

This study demonstrates the prevalence of self-reported kidney disease based on physical activity levels. It was observed that patients with kidney disease were less active as compared to the normal population. In a similar prospective cohort study of 3,926 participants, cardiovascular complications such as heart failure, stroke, and myocardial infarction in patients with CKD over 13.2 years were analyzed, and it was concluded that patients with higher levels of physical activity had lower all-cause mortality (HRs of 0.54) [11]. In another observational cohort study, 450 patients suffering from CKD not on RRT were given a questionnaire to assess their physical activity. It concluded that reduced physical activity increased mortality and the need for RRT [12].

The study also demonstrates the prevalence of self-reported kidney disease and physical activity based on demographic characteristics. It shows that the age group 25-44 years (70.8%), male gender (63.6%), and the White race (59.4%) with kidney disease were more physically active as compared to other study groups. A similar observational study consisting of 5,656 patients with CKD was conducted in England from July 2012 to October 2018. The prevalence of physical activity was low in severe disease with various other comorbidities, older adults, and female gender [13].

In this study, it was also observed that, in comparison to other groups, participants with higher education, employed, and higher annual income were more physically active. A similar cross-sectional study including 512 patients was assessed for quality of life based on physical activity and socio-economic characteristics and concluded that patients with higher educational levels (p=0.008) have better physical and mental quality of life [14]. It was concluded that the prevalence of kidney disease in participants was lower in patients with less than one year of annual routine checks.

This study thus shows that higher physical activity in patients with kidney disease has a higher quality of life and a reduction in the progression of kidney disease, in accordance with the findings by Mallamaci et al. [15].

Limitations

The BRFSS database reported data through a telephone, and the details of the survey were done in English; therefore, a lack of landline or availability during the survey or lack of understanding of the language underestimates the results. The disease is self-reported, so a clinical evaluation by a doctor based on history and examination is not taken into consideration. Also, the type and severity of kidney disease, the evolution of the symptoms, and the complications of kidney disease are not mentioned in the BRFSS database. The physical activity levels were assessed for the past one month, so it is difficult to analyze the prognosis of kidney disease with a shorter duration of physical activity. The study does not precisely compare the onset of kidney disease and the duration of the patients being physically active or inactive.

This observational study is retrospective, so only the association between physical activity and kidney disease is analyzed. However, causation and reduction are not studied, so this can be analyzed with a prospective study.

## Conclusions

The participants with kidney disease who self-reported physical activity were found to have statistical significance between various demographic, socioeconomic, and last routine checkup variables. The data revealed that kidney disease prevalence was significantly lower among physically active patients of all age groups, with male participants reporting more levels of physical activity. Racial disparities showed that White non-Hispanic individuals had the highest prevalence of physical activity levels, followed by other races, whereas Black non-Hispanics showed lower physical activity levels.

Higher-income and advanced education levels were associated with higher levels of physical activity. Employment status also played a key contributor, as those with jobs engaged in greater physical activity than those without employment. Furthermore, there was a significant correlation between the duration since the last regular checkup and physical activity levels; individuals who had a checkup within the previous year were more physically active than those who had not. Further research should aim for a prospective study to evaluate how physical activity modifies kidney disease. Additionally, interventions must be designed to increase physical activity among kidney disease patients with a focus on high-risk demographic groups like older adults, people with lower socioeconomic status, and people who don't get regular checkups.

## References

[1] Liu C, Yang J, Li H (2024). Comparative efficacy of exercise modalities for general risk factors, renal function, and physical function in non-dialysis chronic kidney disease patients: a systematic review and network meta-analysis. Ren Fail.

[2] Tomson CR, Cheung AK, Mann JF (2021). Management of blood pressure in patients with chronic kidney disease not receiving dialysis: synopsis of the 2021 KDIGO clinical practice guideline. Ann Intern Med.

[3] Chatrenet A, Piccoli G, Audebrand JM (2023). Analysis of the rate of force development reveals high neuromuscular fatigability in elderly patients with chronic kidney disease. J Cachexia Sarcopenia Muscle.

[4] Roshanravan B, Gamboa J, Wilund K (2017). Exercise and CKD: skeletal muscle dysfunction and practical application of exercise to prevent and treat physical impairments in CKD. Am J Kidney Dis.

[5] Painter P, Marcus RL (2013). Assessing physical function and physical activity in patients with CKD. Clin J Am Soc Nephrol.

[6] Villanego F, Naranjo J, Vigara LA (2020). Impact of physical exercise in patients with chronic kidney disease: Sistematic review and meta-analysis. Nefrologia (Engl Ed).

[7] Wang AY, March DS, Burton JO (2023). Physical activity and nutrition in chronic kidney disease. Curr Opin Clin Nutr Metab Care.

[8] Noor H, Reid J, Slee A (2021). Resistance exercise and nutritional interventions for augmenting sarcopenia outcomes in chronic kidney disease: a narrative review. J Cachexia Sarcopenia Muscle.

[9] Uchiyama K, Adachi K, Muraoka K (2021). Home-based aerobic exercise and resistance training for severe chronic kidney disease: a randomized controlled trial. J Cachexia Sarcopenia Muscle.

[10] Zhang H, Wang H, Huang L, Bai Y, Zhang F (2023). Interventions to increase physical activity level in patients with whole spectrum chronic kidney disease: a systematic review and meta-analysis. Ren Fail.

[11] Bruinius JW, Hannan M, Chen J (2022). Self-reported physical activity and cardiovascular events in adults with CKD: findings from the CRIC (chronic renal insufficiency cohort) study. Am J Kidney Dis.

[12] Clarke AL, Zaccardi F, Gould DW, Hull KL, Smith AC, Burton JO, Yates T (2019). Association of self-reported physical function with survival in patients with chronic kidney disease. Clin Kidney J.

[13] Wilkinson TJ, Clarke AL, Nixon DG (2021). Prevalence and correlates of physical activity across kidney disease stages: an observational multicentre study. Nephrol Dial Transplant.

[14] Molsted S, Wendelboe S, Flege MM, Eidemak I (2021). The impact of marital and socioeconomic status on quality of life and physical activity in patients with chronic kidney disease. Int Urol Nephrol.

[15] Mallamaci F, Pisano A, Tripepi G (2020). Physical activity in chronic kidney disease and the EXerCise introduction to enhance trial. Nephrol Dial Transplant.

